# Three new species of *Columnea* (Gesneriaceae) from Colombia’s Cordillera Occidental

**DOI:** 10.3897/phytokeys.281.211856

**Published:** 2026-11-06

**Authors:** John L. Clark, Marisol Amaya-Márquez, Oscar Humberto Marín–Gómez, James F. Smith, Laura Clavijo

**Affiliations:** 1 Marie Selby Botanical Gardens, 1534 Mound St., Sarasota, Florida 34236, USA Marie Selby Botanical Gardens Sarasota United States of America; 2 Universidad Nacional de Colombia–Sede Bogotá, Facultad de Ciencias, Instituto de Ciencias Naturales, Bogotá, D.C., 111321, Colombia Universidad Nacional de Colombia–Sede Bogotá, Facultad de Ciencias, Instituto de Ciencias Naturales Bogotá Colombia; 3 Centro de Estudios e Investigaciones en Biodiversidad y Biotecnología (CIBUQ), Universidad del Quindío, Carrera 15 Calle 12 Norte, Armenia, Colombia Centro de Estudios e Investigaciones en Biodiversidad y Biotecnología (CIBUQ), Universidad del Quindío Armenia Colombia; 4 Department of Biology, Boise State University, 1910 University Drive, Boise, Idaho 83725, USA Department of Biology, Boise State University Boise United States of America

**Keywords:** Andes, Colombia, *
Columnea
*, Gesneriaceae, taxonomy

## Abstract

Recent field expeditions in Colombia’s Cordillera Occidental, combined with ongoing herbarium research, have resulted in the discovery of three new species of *Columnea* (Gesneriaceae). These species are described, illustrated with field images, and compared with morphologically similar taxa. The new species are: *Columnea baqueroi* O.H.Marín, J.F.Sm. & J.L.Clark, **sp. nov**.; *Columnea galeanoae* Clavijo, M.Amaya & J.L.Clark, **sp. nov**.; and *Columnea magnicalyx* J.L.Clark, M.Amaya & Clavijo, **sp. nov**.

Resumen

Expediciones de campo recientes a la Cordillera Occidental de Colombia, junto con investigaciones en curso en herbarios, dieron como resultado el descubrimiento de tres nuevas especies de *Columnea* (Gesneriaceae). Estas especies se describen, se ilustran con fotografías de campo y se comparan con taxones morfológicamente similares. Las nuevas especies son: *Columnea baqueroi* O.H.Marín, J.F.Sm. & J.L.Clark, **sp. nov**.; *Columnea galeanoae* Clavijo, M.Amaya & J.L.Clark, **sp. nov**.; y *Columnea magnicalyx* J.L.Clark, M.Amaya & Clavijo, **sp. nov**.

## Introduction

The family Gesneriaceae (Lamiales) comprises more than 3,900 species distributed among 150+ genera (Weber 2004; Weber et al. 2013, 2020; GRC 2026). Current phylogenetic evidence recognizes three subfamilies and seven tribes, all of which are strongly supported as monophyletic lineages (Möller and Clark 2013; Ogutcen et al. 2021). The greatest diversity in the Neotropics occurs within the subfamily Gesnerioideae which includes more than 1,200 species in 77 genera (Clark et al. 2020; GRC 2026). Within Gesnerioideae, *Columnea* L. belongs to the tribe Gesnerieae and subtribe Columneinae (Weber et al. 2013, 2020). One morphological character that distinguishes *Columnea* from closely related genera is the presence of indehiscent berry fruits, in contrast to the fleshy, bivalved capsules characteristic of most other members of the subtribe.

With 234 currently accepted species (GRC 2026), *Columnea* is the largest genus in the Neotropical Gesnerioideae (Weber et al. 2013, 2020; Clark et al. 2020). The genus ranges from southern Mexico to Bolivia, with its center of diversity located in the northern Andes, particularly Colombia and Ecuador. The known diversity of Gesneriaceae in Colombia has increased substantially over the past three decades, from approximately 80 species reported by Kvist et al. (1998), to 95 species reported by Clavijo et al. (2015), and now exceeding 105 species.

Molecular phylogenetic studies have consistently recovered *Columnea* as a monophyletic genus (Clark et al. 2006; Smith et al. 2013; Schulte et al. 2014). In contrast, many of the historically recognized genera now treated within *Columnea*, as well as previously proposed infrageneric classifications, are not supported by phylogenetic studies and are now regarded as artificial (Smith and Carroll 1997; Smith 2000; Clark and Zimmer 2003; Clark et al. 2006, 2012; Smith et al. 2013; Schulte et al. 2014). Consequently, the new species described here are not assigned to any previously recognized infrageneric classification. The species described here illustrate the broad morphological variability of vegetative and floral traits in *Columnea*, including inflorescence reduction, leaf pigmentation, calyx enlargement, and nectary architecture.

## Materials and methods

**Figure 1.**
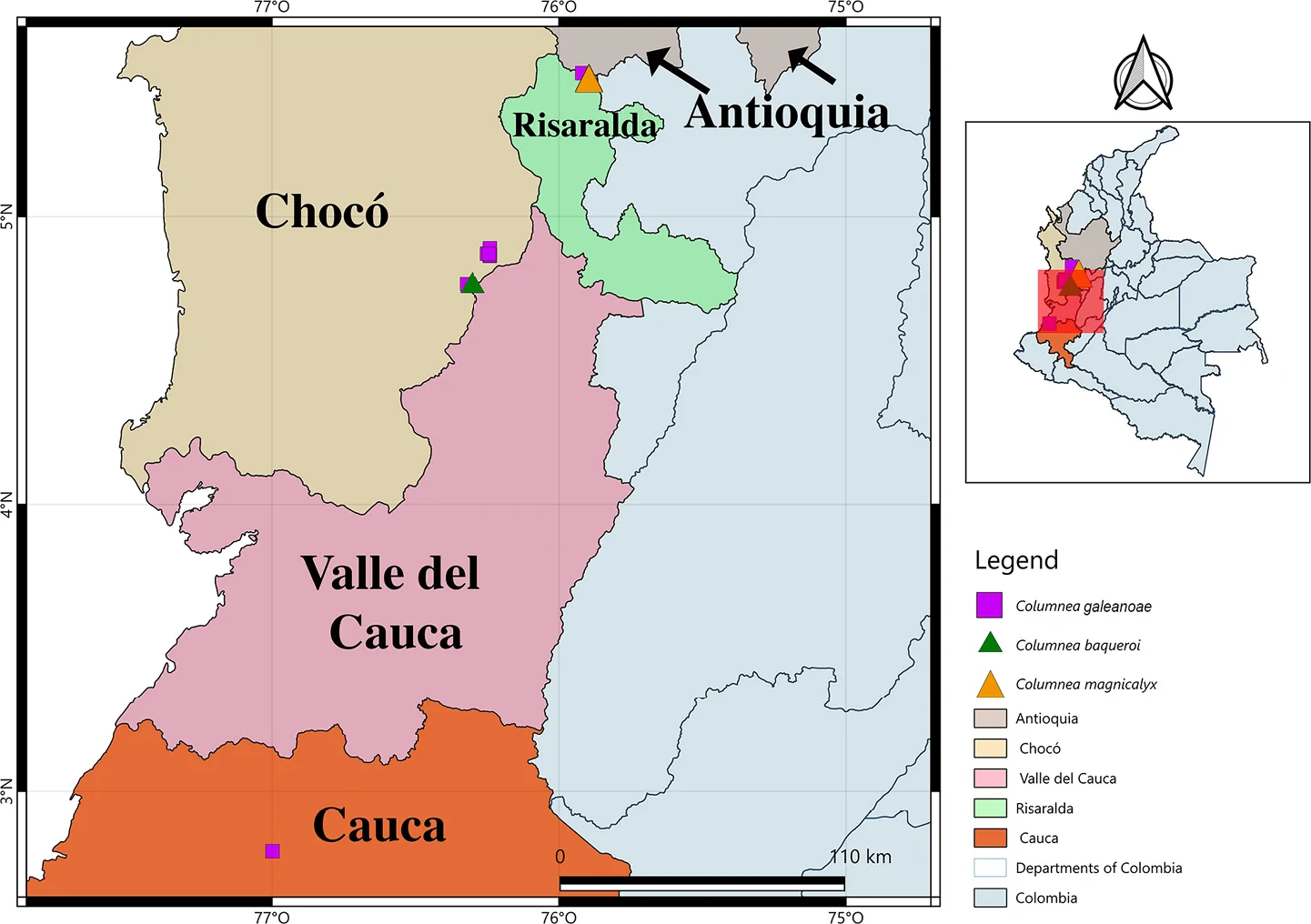
Distribution map for *Columnea baqueroi*, *C. galeanoae*, and *C. magnicalyx*. Map by Wady Martinez.

Specimens representing the three species were documented during ongoing botanical expeditions to the Cordillera Occidental of the Colombian Andes (Fig. 1). *Columnea baqueroi* O.H.Marín, J.F.Sm. & J.L.Clark was first observed in 2011 near San José del Palmar, Chocó, and subsequently documented during a 2024 expedition to the Reserva Natural Comunitaria Cerro El Inglés in the Serranía de los Paraguas. A 2026 expedition to the Mesenia-Paramillo Nature Reserve, managed by Fundación Bioconservancy, resulted in additional collections of *Columnea galeanoae* Clavijo, M.Amaya & J.L.Clark and *Columnea magnicalyx* J.L.Clark, M.Amaya & Clavijo.

Morphological terminology and character-state definitions follow the treatments of Beentje (2016) and Harris and Harris (2006). Photographs of living plants were obtained in the field using a Nikon DSLR camera with a 105 mm macro lens and an SB-29S ring flash. Descriptions and measurements were based on observations of living plants, dried herbarium specimens, alcohol-preserved material, and high-resolution digital images. Measurements from digital images were obtained using ImageJ software (Schneider et al. 2012).

## Taxonomic treatment

### Columnea baqueroi

Taxon classificationPlantaeLamialesGesneriaceae

O.H.Marín, J.F.Sm. & J.L.Clark sp. nov.

3504B992-297A-5D8E-BFEA-628DBAD0D058

urn:lsid:ipni.org:names:77495560-1

Fig. 2

#### Diagnosis.

Distinguished from all other species of *Columnea* by the absence of floral bracts and recurved calyx lobes with gland-tipped, fimbriate margins.

**Figure 2.**
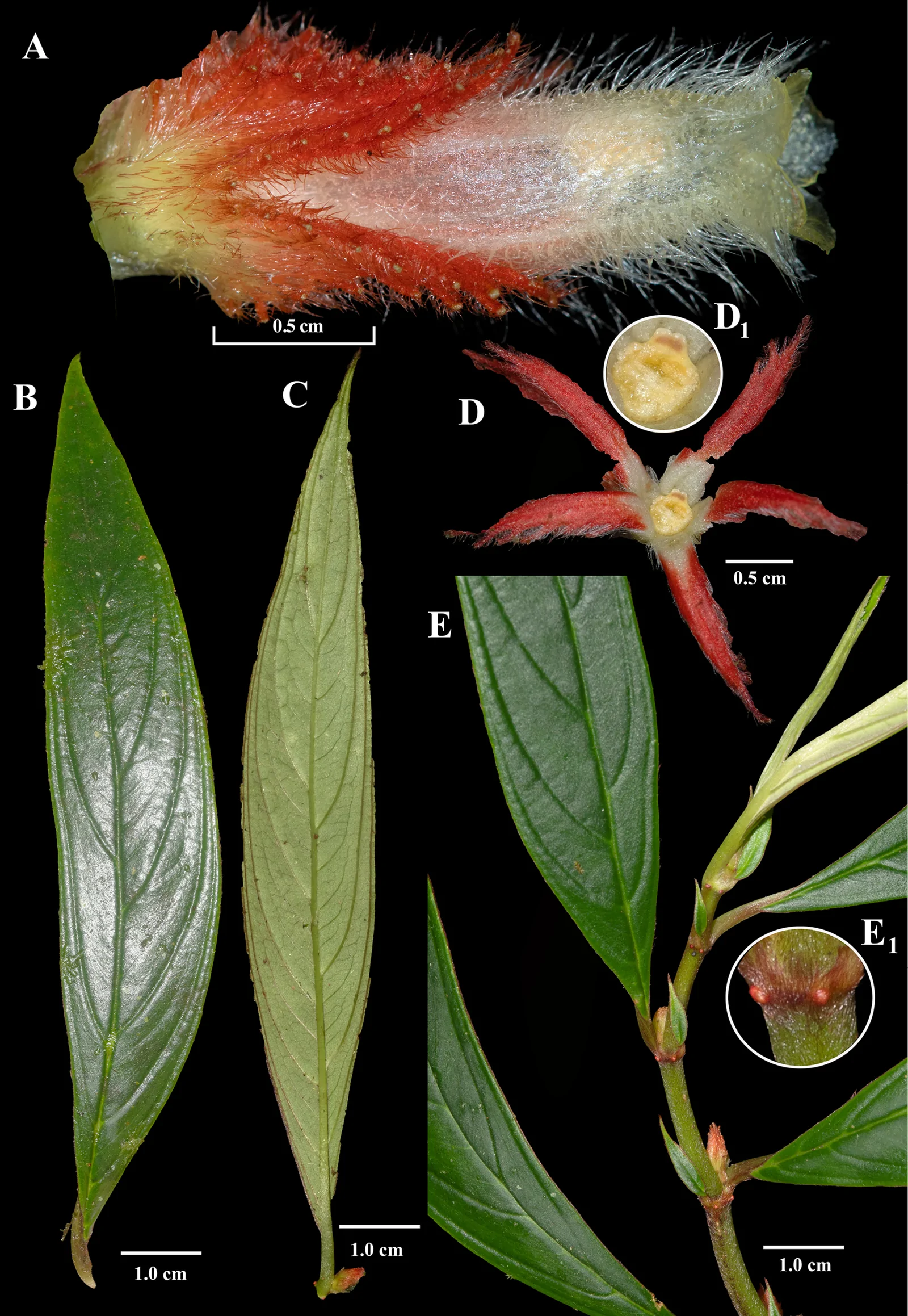
Field images of *Columnea baqueroi* O.H.Marín, J.F.Sm. & J.L.Clark. **A**. Lateral view of flower; **B**. Adaxial leaf surface; **C**. Abaxial leaf surface; **D**. Calyx with inset of bilobed dorsal nectary gland; **E**. Shoot with inset of enations (**A–E** from *J.L. Clark et al. 19474*). Photos by J.L. Clark.

#### Type.

Colombia • Chocó: Municipio San José del Palmar, western slopes of the Cordillera Occidental, Serranía de los Paraguas, Reserva Natural Comunitaria Cerro El Inglés, trail from Santico towards La Florida, 4°45'34.5"N 76°18'03.9"W, 2000–2300 m, 15 August 2024, *J.L. Clark, E. Barco, N. Salinas & L. Clavijo 19474* (holotype: COL!; isotypes: SEL!).

#### Description.

Nomadic climber. ***Stems*** dorsiventral, scandent, branched, terete in cross-section, 3.5–5.5 mm diam., herbaceous to subwoody, green, sparsely pilose apically to glabrescent basally; internodes 2–5 cm long, each node with 2–4 prominent gland-like enations. ***Leaves*** opposite and anisophyllous; petiole of the larger leaf 1–2 cm long, terete, strigose, larger blade narrowly elliptic, 14–20 × 2.2–3.4 cm, membranaceous, dark green and glabrous adaxially, light green and uniformly pilose abaxially, apex acute to acuminate, base cuneate, margin entire, blades with 6–8 pairs of secondary veins; smaller blade sessile, linear to lanceolate, 0.8–1.1 × 0.3–0.4 cm, 2–3 pairs of lateral veins, often caducous. ***Inflorescence*** reduced to 1–4 axillary flowers; peduncles and bracts absent; pedicels 1–3 mm long, green, sparsely pilose. ***Calyx*** lobes 5, fused basally for 1–2 mm, equal in length, white at base on inner surface, otherwise uniformly red on outer and inner surfaces, 1.2–1.4 × 0.2–0.4 cm, narrowly elliptic to linear, margins recurved lengthwise, apex acute, base truncate, margin fimbriate, each fimbria green and glandular at the apex, densely pilose on the outer surface, glabrous on the inner surface. ***Corolla*** uniformly tubular, 1.9–2.2 cm long; tube erect relative to calyx, 3.5–4.0 mm wide, uniformly white and often translucent, densely pilose, corolla with 5 equal-sized lobes, 1.8–1.9 × 2.1–2.4 mm, reflexed during anthesis, apex rounded, margin entire, densely pilose abaxially. ***Androecium*** of 4 didynamous stamens, filaments to 2 cm long, adnate to the corolla tube for 1.5–2.0 mm, glabrous, staminode absent; anthers oblong, coherent, dehiscing by longitudinal slits, 2.2–2.5 × 2.4–2.6 mm. ***Gynoecium*** with a bilobed dorsal nectary gland, ca. 2 mm long, glabrous; ovary superior, ca. 4 × 3 mm, ovate, sparsely pilose; style included, ca. 1.8 cm long, glabrous; stigma bilobed. ***Fruit*** not observed.

#### Phenology.

Collected with flowers in August. Fruit not observed.

#### Etymology.

The specific epithet *baqueroi* honors the Colombian botanist Julio César Baquero Rojas (1989–2025) in recognition of his passion for the study and conservation of Andean epiphytes. We dedicate this species to his memory as both a valued colleague and friend.

#### Distribution and conservation status.

*Columnea baqueroi* is currently known only from mature montane cloud forests in the Serranía de los Paraguas, within the Reserva Natural Comunitaria Cerro El Inglés, on the western slopes of the Cordillera Occidental in the Chocó Department of Colombia (Fig. 1). The species was documented along a forest trail within the reserve. The reserve is part of a community-based conservation initiative supported by Corporación Serraniagua, a Colombian nonprofit organization that works with local communities to protect montane forests and promote sustainable land management in the Serranía de los Paraguas. Although the occurrence of *C. baqueroi* within a protected area provides habitat protection, its apparently restricted distribution and limited number of documented collections warrant further field surveys to determine its geographic range, population size, and conservation status. A preliminary conservation assessment is premature because available data are insufficient to estimate population size, geographic range, and potential threats throughout the species’ distribution.

#### Notes.

*Columnea baqueroi* is readily recognized by the combination of reduced inflorescences that appear as axillary clusters of flowers lacking floral bracts, and recurved calyx lobes with fimbriate margins, each fimbria terminating in a green, gland-like apex (Fig. 2A). The absence of floral bracts is uncommon in *Columnea*. Prominently gland-tipped, fimbriate calyx margins are known from only a few other species, including *Columnea laciniata* J.L.Clark & M.Amaya and *C. versicolor* J.L.Clark & Clavijo. The white, tubular corolla, with only weakly developed bilabiate lobes, is also unusual in the genus, where brightly colored, strongly bilabiate corollas predominate. The narrowly elliptic leaves further contribute to the distinctive appearance of the species (Fig. 2 B, C). In addition, the nodes bear prominent enations (nectary-like glands) situated between the opposing leaf bases (Fig. 2E). Although stem enations are present in most members of the subtribe Columneinae, they are rarely as conspicuous as those observed in *C. baqueroi*.

### Columnea galeanoae

Taxon classificationPlantaeLamialesGesneriaceae

Clavijo, M.Amaya & J.L.Clark sp. nov.

BA36DD51-83C7-5B71-88B8-1FEC4EBAE587

urn:lsid:ipni.org:names:77495561-1

Figs 3, 4

#### Diagnosis.

Differs from all other congeners by its prominent pilose indumentum on vegetative surfaces and dark purple to red abaxial leaf surfaces with green apices (vs. green abaxial surfaces with red apices). The prominent pilose indumentum resembles that of *C. ciliata*, but the two species differ in corolla and fruit morphology: *C. ciliata* has laterally compressed corolla tubes and ovoid berries, whereas *C. galeanoae* has rounded (not laterally compressed) corolla tubes and oblong berries.

**Figure 3.**
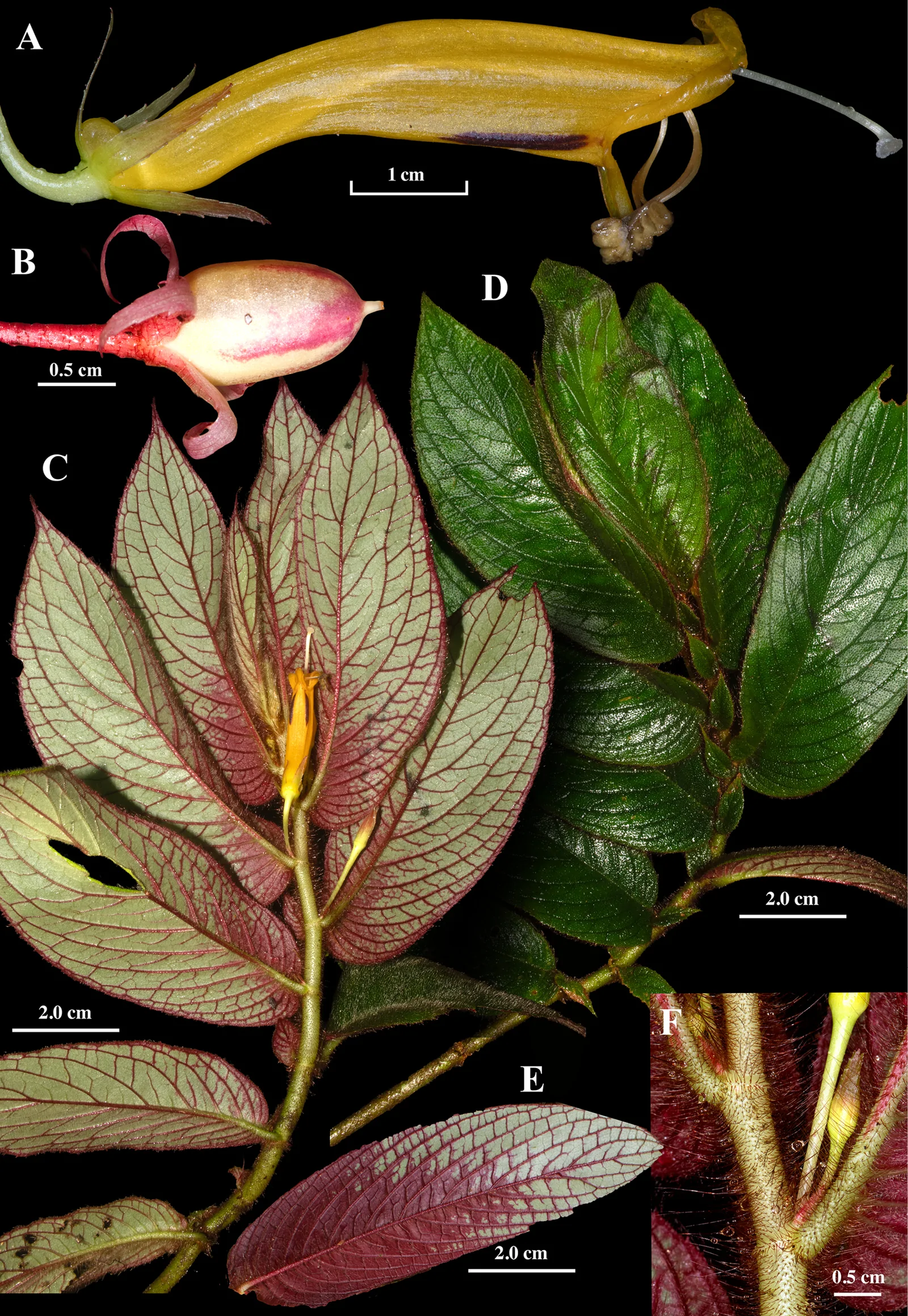
Field images of *Columnea galeanoae* Clavijo, M.Amaya & J.L.Clark. **A**. Lateral view of flower; **B**. Mature fruit; **C**. Habit showing abaxial leaf surfaces; **D**. Habit showing adaxial leaf surfaces; **E**. Habit showing abaxial leaf surface with red pigmentation basally and green apically; **F**. Stem and petioles featuring pilose indumentum (**A, C–F** from *J.L. Clark et al. 20603*; **B** from *L. Clavijo et al. 1889*). Photos by J.L. Clark.

**Figure 4.**
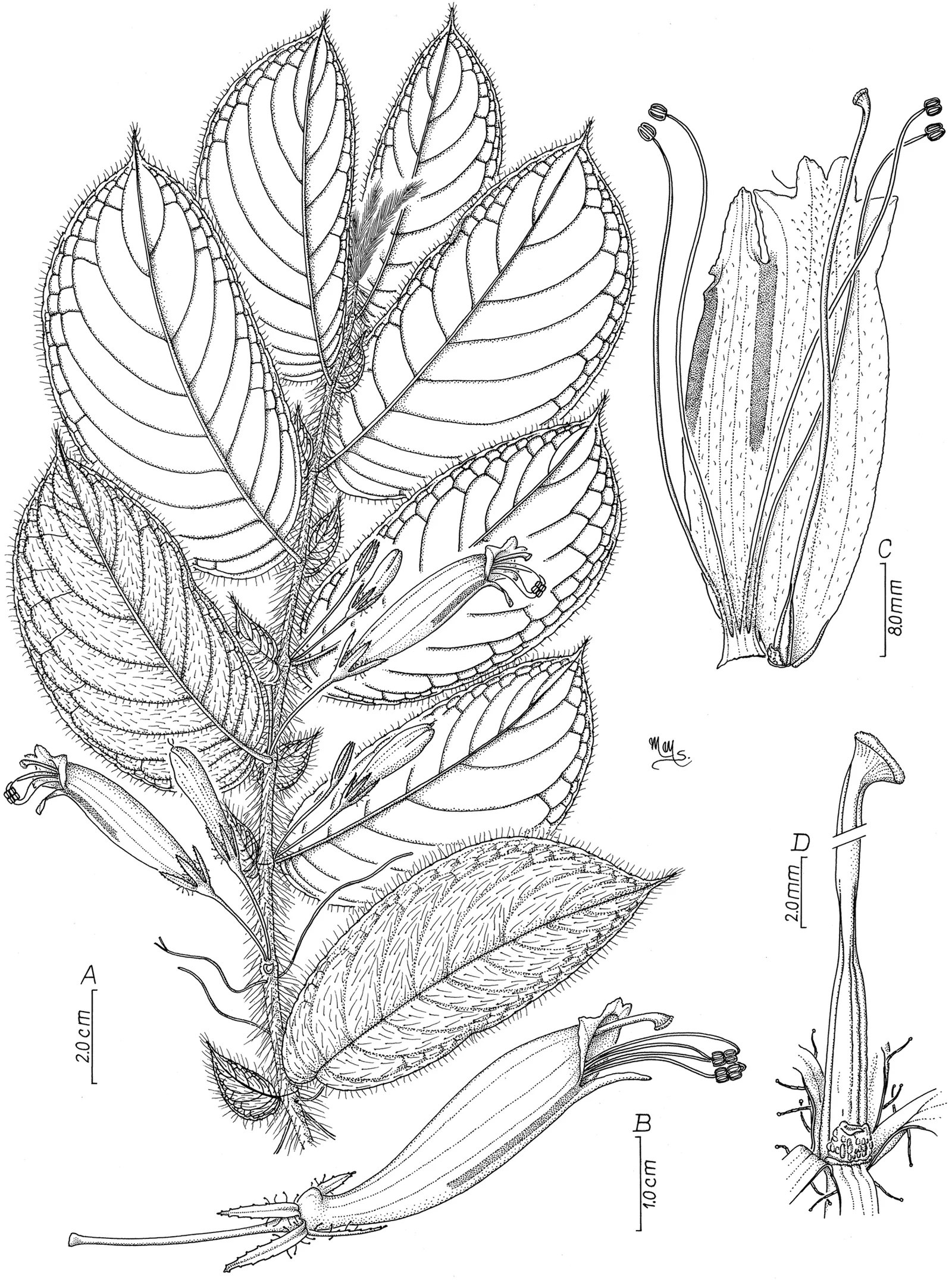
Illustration of *Columnea galeanoae* Clavijo, M.Amaya & J.L.Clark. **A**. Habit; **B**. Lateral view of flower; **C**. Open flower; **D**. Gynoecium. Illustration by Marcela Morales and based on *L. Clavijo et al. 1759*.

#### Type.

Colombia • Chocó: Municipio San José del Palmar, road from Ansermanuevo to San José del Palmar, San Antonio village, trail leading to Las Cascadas stream and Mina Rica, 4.847750, -76.226528, 1100–1600 m, 9 Aug 2011, *L. Clavijo, A. Zuluaga, Ó.H. Marín Gómez & M. Pineda 1782* (holotype: COL!).

#### Description.

Nomadic climber. ***Stems*** dorsiventral, scandent, branched, subquadrate in cross-section, 4–8 mm in diam., suffrutescent, becoming red distally, basally sparsely to densely pilose; internodes 1.5–5.5 cm long. ***Leaves*** opposite and anisophyllous; petiole of the larger leaf 0.2–0.7 cm long, densely pilose; blade broadly oblong to elliptic, 8–30 × 3.7–9 cm, membranaceous to chartaceous, adaxial surface uniformly green and pilose, abaxial surface dark purple to red basally, becoming pale green apically, uniformly pilose, base asymmetrical and cordate, margin irregularly serrate, blades with 8–13 pairs of secondary veins, tertiary veins reticulate; smaller blade with petiole 0.2–0.4 cm long, blade narrow ovate to lanceolate, 2–4 × 1–1.3 cm, adaxial surface uniformly green and pilose, abaxial surface uniformly deep purple and uniformly pilose, base asymmetrical and cordate, margin irregularly serrate, blades with 4–5 pairs of lateral veins. ***Inflorescence*** reduced to 1–4 axillary flowers; peduncles and bracts absent; pedicels 1.7–3 cm long, thickened and recurved distally, reddish, glabrous, with scattered small enations. ***Calyx*** lobes 5, lobes fused basally for 1–2 mm, subequal in length, white on inner surface, outer surface green or red, 0.8–1.1 × 0.2–0.3 cm, lanceolate, margins dentate, outer surface with uniseriate multicellular glandular trichomes, glabrous on the inner surface. ***Corolla*** tubular, 4.2–5.5 cm long; tube erect relative to calyx, with a basal spur 0.35–0.4 × 0.4–0.6 cm, limb distally bilabiate with 5 unequal-sized lobes, upper lip formed by two dorsal obtuse lobes and two lateral triangular lobes connate nearly to the apex, lip 1.1–1.3 × 0.9–1.1 cm, ventral lobe oblong and reflexed at anthesis forming the lower lip 0.8 × 0.1–0.2 cm; corolla outside yellow with two prominent deep purple lines running from below the ventral lobe toward the middle of the tube; glabrous outside, sparsely pubescent with glandular trichomes clustered on the dorsal lobes inside. ***Androecium*** of 4 didynamous stamens, filaments 4.0–5.2 cm long, adnate to the corolla tube for 2.5 cm, glabrous, staminode absent; anthers rectangular, coherent, dehiscing by longitudinal slits, 1.5 × 1 mm. ***Gynoecium*** with a dorsal nectary gland, ca. 1.5 mm long; ovary superior, ca. 6 × 1.5 mm, oblong, glabrous; style exserted, ca. 4.0–4.2 cm long, glabrous; stigma discoid. ***Fruit*** an oblong berry, white with purple stripes or flushed purple, 1.8 × 0.9 cm.

#### Additional specimens examined.

Colombia • Cauca: El Tambo, Parque Nacional Munchique, camino a Lopez de Micay, entre El Boquerón y La Cueva, 2°46'5.4"N, 76°58'27.7"W, 900–1050 m, 15 June 2017, *L. Clavijo et al. 1889* (COL). • Chocó: Municipio San José del Palmar, Serranía de los Paraguas, western slopes of the Cordillera Occidental, sector San Antonio, road towards San José del Palmar, near the departmental border of Valle del Cauca, 4°50'24.7"N, 76°13'1.42"W, 1650–1750 m, 11 August 2024, *J.L. Clark et al. 19292* (COL, SEL); • Municipio San José del Palmar, carretera de Ansermanuevo a San José del Palmar, vereda San Antonio, camino a Las Cascadas, 4°50'32.3"N, 76°12'58.6"W, 1600–1710 m, 8 August 2011, *L. Clavijo et al. 1759* (COL); • Municipio San José del Palmar, vereda San Antonio, Escuela San Antonio, 4°52'N, 76°13'W, 1300 m, 13 May 2011, *O.H. Marín-Gómez & A. Gómez-Hoyos 85* (COL); • Municipio San José del Palmar y Cairo, sector La Florida, camino Los Santicos la Florida, Reserva Natural Cerro del Inglés, 4.7417°N, 76.2962°W, 2177 m, 20 May 2013, *J.F. Smith et al. 10843* (COL, SRP); • Municipio San José del Palmar, camino hacia el río Habita, 4°50.845'N, 76°13.174'W, 1100–1700 m, 25 May 2013, *J.F. Smith et al. 10928* (COL, SRP). • Risaralda: Municipio Mistrató, Reserva Natural Mesenia-Paramillo (Fundación Bioconservancy), trail from El Alto to Descanso de La Madre Laura, near Río San Juan Bravo, 5°28'27.81"N, 75°53'41.77"W, 1900–2200 m, 24 March 2026, *J.L. Clark & U. Rendón 20603* (COL, JAUM, SEL).

#### Phenology.

Collected with flowers and fruit in March, May, June, and August.

#### Etymology.

The specific epithet *galeanoae* honors the late Colombian botanist Gloria Galeano Garcés (1958–2016), in recognition of her outstanding contributions to the taxonomy, ecology, and conservation of the Colombian flora, particularly her pioneering studies of Neotropical palms. The type collection was made by her former students as a tribute to her enduring legacy as a scientist and mentor.

#### Distribution and conservation status.

*Columnea galeanoae* is endemic to the western slopes of the Cordillera Occidental of Colombia, where it occurs in mature montane forests in the departments of Cauca, Chocó, and Risaralda (Fig. 1), at elevations ranging from 900 to 2,200 m. In Cauca, the species is documented from Parque Nacional Natural Munchique; in Chocó, from the Serranía de los Paraguas near San José del Palmar, including Reserva Natural Comunitaria Cerro El Inglés; and in Risaralda, from the Mesenia-Paramillo Nature Reserve. These localities encompass both publicly and privately protected areas, including conservation initiatives supported by Corporación Serraniagua, which promotes community-based forest conservation in the Serranía de los Paraguas, and Fundación Bioconservancy, which manages the Mesenia-Paramillo Nature Reserve. Although several documented populations occur within protected areas, additional field surveys are needed to assess the species’ full geographic distribution, population size, and conservation status.

#### Comments.

In *Columnea galeanoae*, the abaxial leaf surface exhibits a striking inverted pattern of pigmentation, with dark purple to red pigmentation extending from the base over most of the blade, while the apex remains green (Fig. 3C, E). Nearly all species of *Columnea* with anisophyllous leaves have leaf blades that are green on the abaxial surface, with red pigmentation confined to the apical portion of the blade. *Columnea galeanoae* represents only the second known exception to this pattern, the other being *C. eubracteata* Mansf. *Columnea eubracteata* is readily distinguished from *C. galeanoae* by the presence of dense clusters of red floral bracts that largely conceal the calyx lobes and most of the corolla, whereas floral bracts are absent in *C. galeanoae*.

The species is further distinguished by conspicuous, densely pilose red trichomes (Fig. 3F), particularly on the stems and petioles. In addition, the inflorescences are highly reduced, consisting of one to four flowers per leaf axil (Figs 3, 4). The larger leaf of the anisophyllous pair exhibits considerable variation in size. Plants from higher-elevation populations in the departments of Risaralda and Chocó (1,600–2,200 m) have relatively smaller leaves (8–10 × 3.7–4.2 cm), whereas those from lower-elevation populations in the department of Cauca (900–1,050 m) have larger leaf blades (20–30 × 4.2–9.0 cm). Despite this variation, these populations are characterized by dense indumentum on the stems and leaves and a glabrous corolla tube.

### Columnea magnicalyx

Taxon classificationPlantaeLamialesGesneriaceae

J.L.Clark, M.Amaya & Clavijo sp. nov.

6F7D7A1F-A930-590A-9F58-E3678100FE5B

urn:lsid:ipni.org:names:77495562-1

Fig. 5

#### Diagnosis.

Distinguished from all other species of *Columnea* by the combination of large calyx lobes that exceed half the length of the corolla tube, solitary axillary flowers, a four-lobed nectary surrounding the ovary, and prominent red pilose trichomes on the stems, petioles, and leaf margins.

**Figure 5.**
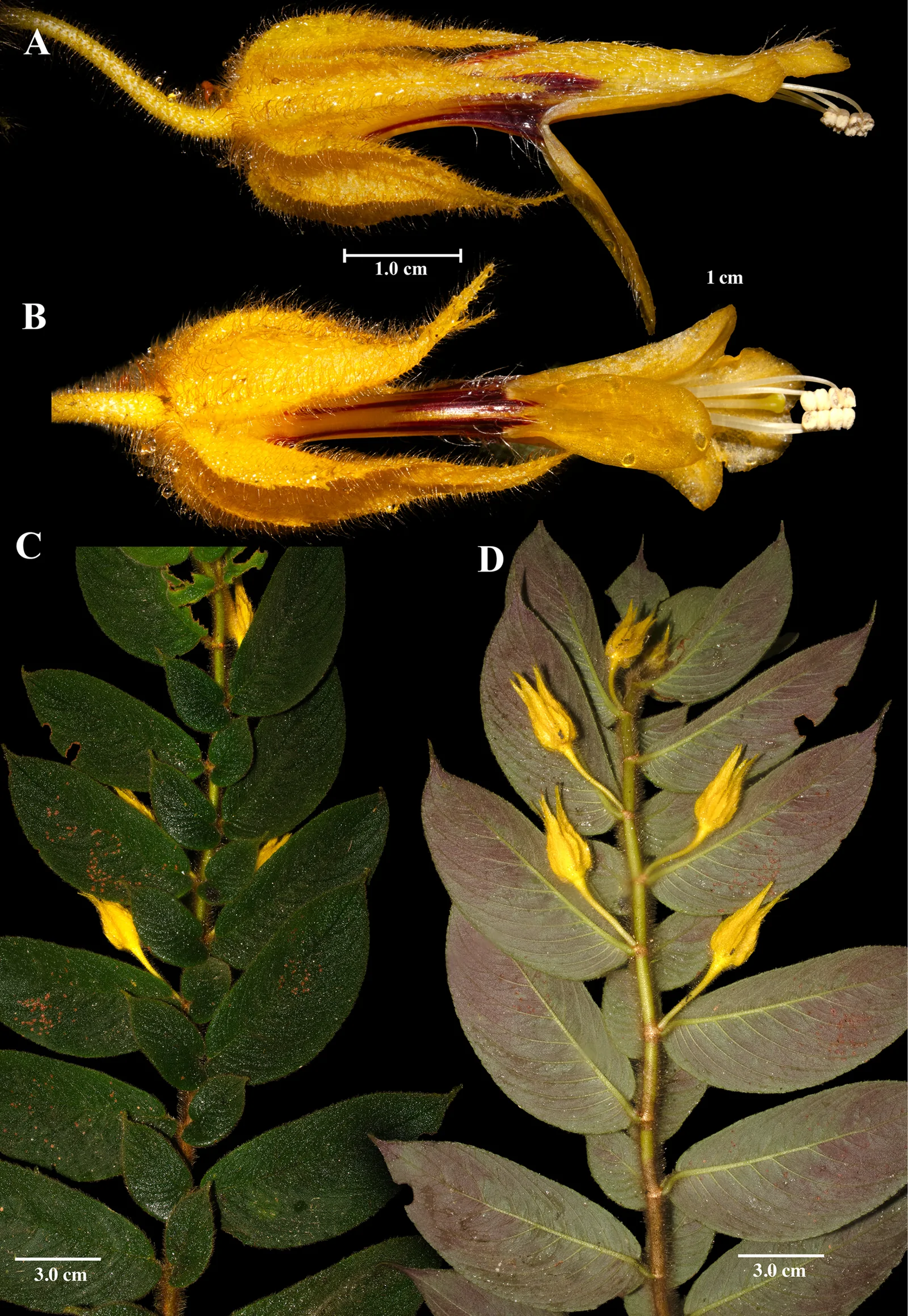
Field images of *Columnea magnicalyx* J.L.Clark, M.Amaya & Clavijo. **A**. Lateral view of mature flower; **B**. Dorsal view of mature flower; **C**. Adaxial habit view; **D**. Abaxial habit view (**A, B** from *L. Clavijo et al. 2671*; **C, D** from *J.L. Clark et al. 20564*). Photos (**A, B**) by L. Clavijo; photos (**C, D**) by J.L. Clark.

#### Type.

Colombia • Risaralda: Municipio Mistrató, Cordillera Occidental, path from Río San Juan to San Antonio de Chamí, 5°28'58"N 75°53'42"W, 2200–2500 m, 17 May 2012, *J.L. Clark, J. Anderson, L. Clavijo & U. Rendón 12978* (holotype: COL!; isotypes: SEL! [barcode SEL077584]).

#### Description.

Nomadic climber. Stems dorsiventral, scandent, branched, terete in cross-section, 3–5 mm diam., herbaceous to subwoody, green, uniformly densely pilose; internodes 2–4 cm long. ***Leaves*** opposite and anisophyllous; larger leaf with petiole 3.0–4.4 mm long, terete, pilose; blade elliptic, 8–13 × 3–5 cm, coriaceous, dark green and densely pilose adaxially, light green, sometimes suffused with red, and sparsely pilose abaxially; apex acute, base oblique and rounded, margin shallowly serrulate; secondary veins 10–13 pairs; smaller leaf sessile; blade ovate-elliptic, 2–4 × 1.5–2.0 cm; secondary veins 6–8 pairs; abaxial and adaxial surfaces otherwise similar to those of the larger leaf. ***Inflorescence*** reduced to a single axillary flower; bracts absent; pedicel to 3.2 cm long, densely pilose. ***Calyx*** of 5 lobes, fused basally for 1–2 mm; lobes equal, 2.9–3.5 × 0.9–1.5 cm, conduplicate, appressed against adjacent lobes and appearing cucullate, broadly oblong in the proximal half, gradually tapering into a narrow acuminate apex, margins serrate, uniformly yellow, densely pilose externally and glabrous internally. ***Corolla*** strongly bilabiate, tube narrow and laterally compressed at the base, gradually expanding toward the apex, 5.0–6.0 cm long; tube erect relative to the calyx, ca. 8 mm wide at the base of the lower lip, mostly yellow with a prominent pair of horizontal red striations on the ventral surface extending from the base of the tube to the lower lip; upper and lateral lobes fused into an erect galea during anthesis; lower lip reflexed during anthesis; galea ca. 2.0 cm long; lateral lobes and upper lobe entire, lateral lobes ca. 2 × 3 mm, upper lobe ca. 2 × 4.5 mm; lower lip ca. 10 × 3.4 mm. ***Androecium*** of 4 didynamous stamens; filaments more than 5 cm long, exceeding the corolla tube during anthesis, adnate to the corolla tube for ca. 3.5 mm, forming a filament curtain, glabrous; staminode present, ca. 2 mm long; anthers oblong, coherent, ca. 2.5 × 2.3 mm, dehiscing by longitudinal slits. ***Gynoecium*** with a four-lobed nectary surrounding the ovary, dorsal gland larger and apically truncate, the remaining three glands of similar size, glabrous; ovary superior, ca. 4.5 × 3 mm, ovate, sparsely pilose; style ca. 5.0 cm long, included, glabrous; stigma stomatomorphic. Fruit not observed.

#### Additional specimens examined.

Colombia • Risaralda: municipio Mistrató, Reserva Natural Mesenia-Paramillo (Fundación Bioconservancy), trail from MPNR to El Alto and continuing toward Cerro Paramillo, 5°28'59.739"N, 75°53'42.835"W, 2500–2800 m, 23 March 2026, *J.L. Clark, S.E. Hoyos-Gómez, G. Wahlert, S. Clark, A. Clark, A. Suárez & U. Rendón 20564* (COL, JAUM, SEL). • Jardín–Mistrató, on the border between the departments of Antioquia and Risaralda, Reserva Natural Mesenia-Paramillo, trail between the cabin and San Antonio del Chamí, between Alto del Chamí and “Care Perro,” 5°28'45.5"N, 75°53'43.2"W, 2436 m, 9 Aug 2022, *L. Clavijo et al. 2671* (COL, JAUM, CUVC, MO). • Antioquia: municipio Jardín, vereda la Mesenia, Reserva Natural Mesenia-Paramillo, 1800–2300 m, 21 Sep 2012, *J.P. Tobón et al. 338* (JAUM).

#### Phenology.

Collected with flowers in March, May, August, and September. Fruit not observed.

#### Etymology.

The specific epithet *magnicalyx* combines the Latin *magnus* (“great” or “imposing”) with the Greek *kalyx* (“calyx”), alluding to the remarkably large and conspicuous calyx lobes (Fig. 5) that readily distinguish this species from its congeners.

#### Distribution and conservation status.

*Columnea magnicalyx* is currently known only from mature montane cloud forests in the Cordillera Occidental of western Colombia, within the Mesenia-Paramillo Nature Reserve (Fig. 1). This 3,700-ha privately protected area is managed by Fundación Bioconservancy, a nonprofit conservation organization, and is located near Jardín, at the junction of the Antioquia, Caldas, and Risaralda departments, spanning elevations from 1,560 to 3,167 m. Although the occurrence of *C. magnicalyx* within a privately protected reserve provides habitat protection, its apparently restricted distribution and limited number of documented collections warrant further field surveys to determine its geographic range, population size, and conservation status. A preliminary conservation assessment is premature because available data are insufficient to estimate population size, geographic range, and potential threats throughout the species’ distribution.

#### Notes.

*Columnea magnicalyx* is readily recognized by the combination of absent floral bracts, solitary axillary flowers (Fig. 5 C, D), conspicuous red pilose trichomes on the stems, petioles, and leaf margins, and exceptionally large calyx lobes that often exceed half the length of the corolla tube (Fig. 5 A, B). Another remarkable feature is the presence of a four-lobed nectary. Most species of *Columnea* possess a single dorsal nectary gland or a truncate dorsal gland, whereas *C. magnicalyx* has a conspicuous nectary divided into four lobes, with the dorsal lobe larger and truncate.

The calyx lobes are substantially larger than those of most species of *Columnea*, particularly among taxa with anisophyllous leaves. In anisophyllous species, showy inflorescence bracts are common, whereas the calyx lobes are typically inconspicuous and much reduced. In contrast, *C. magnicalyx* lacks floral bracts but has greatly enlarged, showy calyx lobes (Fig. 5). The absence of floral bracts and the enlargement of the calyx may represent alternative morphological strategies for producing a conspicuous floral display.

Floral nectaries have long been considered taxonomically informative in *Columnea* and related genera. Although nectary morphology was used extensively in traditional classifications, most infrageneric groups are not supported by recent phylogenetic studies and are now regarded as artificial (Smith and Carroll 1997; Smith 2000; Clark and Zimmer 2003; Clark et al. 2006, 2012; Smith et al. 2013; Schulte et al. 2014). Wiehler (1983) regarded nectary morphology as a stable and diagnostic character and used it to justify the recognition of sections *Pentadenia* and *Stygnanthe* as the separate genus *Pentadenia*. He also considered the presence of five separate nectary glands to represent the most “primitive” nectary type in tribe Episcieae (= Columneinae in the current classification). In contrast, Kvist and Skog (1993), who adopted a broader circumscription of *Columnea*, documented extensive variation in nectary morphology within the genus, particularly in *Columnea strigosa* Benth., and interpreted this diversity as intraspecific and intrageneric variation rather than evidence supporting generic segregation. In most species of *Columnea*, the nectary consists of a single dorsal gland that is entire, truncate, or occasionally bilobed. One notable exception occurs in species traditionally assigned to sections *Pentadenia* and *Stygnanthe*, in which the nectary comprises three to five lobes forming a ring around the ovary. These species also typically possess corollas with shallowly bilabiate to nearly actinomorphic limbs. In contrast, *Columnea magnicalyx* possesses a conspicuous four-lobed nectary but retains a deeply bilabiate corolla, a floral morphology more characteristic of species traditionally assigned to sections *Collandra* and *Columnea*. The combination of a deeply bilabiate corolla and a multilobed nectary represents an unusual combination of character states in *Columnea* and is otherwise known only in *C. strigosa* Benth.

## Supplementary Material

XML Treatment for Columnea baqueroi

XML Treatment for Columnea galeanoae

XML Treatment for Columnea magnicalyx

## Additional information

### Artificial Intelligence (AI) use

The authors accept full responsibility for the content of the manuscript, including the disclosure of any use of AI.

No AI tools were used in the preparation of this manuscript.

### Author ORCIDs

J.L. Clark https://orcid.org/0000-0002-1414-6380

M. Amaya-Márquez https://orcid.org/0000-0001-6135-8063

O.H. Marín–Gómez https://orcid.org/0000-0002-0743-8350

J.F. Smith https://orcid.org/0009-0007-1870-1277

L. Clavijo https://orcid.org/0000-0002-3009-9158

### Data availability

All of the data that support the findings of this study are available in the main text.
